# Major causes of death in preterm infants in selected hospitals in Ethiopia (SIP): a prospective, cross-sectional, observational study

**DOI:** 10.1016/S2214-109X(19)30220-7

**Published:** 2019-07-11

**Authors:** Lulu M Muhe, Elizabeth M McClure, Assaye K Nigussie, Amha Mekasha, Bogale Worku, Alemayehu Worku, Asrat Demtse, Beza Eshetu, Zemene Tigabu, Mahlet A Gizaw, Netsanet Workneh, Abayneh Girma, Mesfin Asefa, Ramon Portales, Tiruzer Bekele, Mesele Bezabih, Gesit Metaferia, Mulatu Gashaw, Bewketu Abebe, Hailu Berta, Addisu Alemu, Tigist Desta, Rahell Hailu, Goitom Gebreyesus, Sara Aynalem, Alemseged L Abdissa, Riccardo Pfister, Zelalem Tazu Bonger, Solomon Gizaw, Tamrat Abebe, Melkamu A Berhane, Yonas Bekuretsion, Sangappa Dhaded, Janna Patterson, Robert L Goldenberg

**Affiliations:** a College of Health Sciences, Addis Ababa University, Addis Ababa, Ethiopia; b Social, Statistical and Environmental Health Sciences, Research Triangle Institute, Durham, NC, USA; c Bill & Melinda Gates Foundation, Seattle, WA, USA; d Ethiopian Pediatric Society, Addis Ababa, Ethiopia; e Jimma University, Jimma, Ethiopia; f University of Gondar, Gondar, Ethiopia; g St Paul's Hospital Millennium Medical College, Addis Ababa, Ethiopia; h Department of Obstetrics and Gynecology, Columbia University, New York, NY, USA; i Global Child Health and Life Support, American Academy of Pediatrics, Itasca, IL, USA; j Zewditu Hospital, Addis Ababa, Ethiopia; k Neonatology Unit, Hôpitaux Universitaires de Genève, Geneva, Switzerland; l Women's and Children's Health Research Unit, KAHER, J N Medical College, Belgaum, India

## Abstract

**Background:**

Neonatal deaths now account for 47% of all deaths in children younger than 5 years globally. More than a third of newborn deaths are due to preterm birth complications, which is the leading cause of death. Understanding the causes and factors contributing to neonatal deaths is needed to identify interventions that will reduce mortality. We aimed to establish the major causes of preterm mortality in preterm infants in the first 28 days of life in Ethiopia.

**Methods:**

We did a prospective, cross-sectional, observational study in five hospitals in Ethiopia. Study participants were preterm infants born in the study hospitals at younger than 37 gestational weeks. Infants whose gestational age could not be reliably estimated and those born as a result of induced abortion were excluded from the study. Data were collected on maternal and obstetric history, clinical maternal and neonatal conditions, and laboratory investigations. For neonates who died of those enrolled, consent was requested from parents for post-mortem examinations (both complete diagnostic autopsy and minimally invasive tissue sampling). An independent panel of experts established the primary and contributory causes of preterm mortality with available data.

**Findings:**

Between July 1, 2016, to May 31, 2018, 4919 preterm infants were enrolled in the study and 3852 were admitted to neonatal intensive care units. By 28 days of post-natal age, 1109 (29%) of those admitted to the neonatal intensive care unit died. Complete diagnostic autopsy was done in 441 (40%) and minimally invasive tissue sampling in 126 (11%) of the neonatal intensive care unit deaths. The main primary causes of death in the 1109 infants were established as respiratory distress syndrome (502 [45%]); sepsis, pneumonia and meningitis (combined as neonatal infections; 331 [30%]), and asphyxia (151 [14%]). Hypothermia was the most common contributory cause of preterm mortality (770 [69%]). The highest mortality occurred in infants younger than 28 weeks of gestation (89 [86%] of 104), followed by infants aged 28–31 weeks (512 [54%] of 952), 32–34 weeks (349 [18%] of 1975), and 35–36 weeks (159 [8%] of 1888).

**Interpretation:**

Three conditions accounted for 89% of all deaths among preterm infants in Ethiopia. Scale-up interventions are needed to prevent or treat these conditions. Further research is required to develop effective and affordable interventions to prevent and treat the major causes of preterm death.

**Funding:**

Bill & Melinda Gates Foundation.

## Introduction

Every year, an estimated 15 million infants are born preterm (before 37 completed weeks of gestation), and this number is increasing.^1^, ^2^ Preterm infants are susceptible to many complications, including respiratory distress syndrome, chronic lung disease, intestinal injury, compromised immune systems, and cardiovascular disorders.^3^ In 2017, 2·5 million newborn deaths occurred globally, accounting for 47% of deaths in children younger than 5 years.^4^ Slightly more than a third of these deaths resulted from preterm-related causes.^4^ Complications of preterm birth are the leading cause of mortality in children younger than 5 years (18%).^5^, ^6^ With the increasing contribution of neonatal deaths to overall child mortality, there is a need to establish the causes of preterm mortality.^7^, ^8^

Globally, from 1990 to 2016, mortality in children younger than 5 years was reduced by 56% and neonatal mortality by 49%.^6^ In Ethiopia, mortality in children younger than 5 years was reduced from 205 deaths per 1000 livebirths in 1990 to 64 deaths per 1000 livebirths by 2013.^9^ However, there has been slower progress in reducing neonatal mortality (55 deaths per 1000 livebirths in 1990 *vs* 28 deaths per 1000 livebirths in 2013).^9^ Prematurity-related complications have contributed to about 30% of neonatal deaths in Ethiopia.^9^

**Research in context**

**Evidence before this study**

Prematurity was once considered as one condition in the global disease classification nomenclature. Now we know that preterm mortality is responsible for nearly 18 deaths per 1000 livebirths according to the 2018 WHO estimation. As effective interventions become available to prevent some of the specific causes of preterm-related mortality in low-resource settings, there is a need to establish the distribution of the major causes. The causes of mortality in preterm babies include sepsis, asphyxia, respiratory distress syndrome or hyaline membrane disease, cold injury, intraventricular haemorrhage, necrotising enterocolitis, metabolic and electrolyte disturbances, and congenital disorders such as major congenital heart malformations and neurological malformations. However, it is not known which of these conditions contribute to what proportion of preterm mortality. A systematic review of the literature on the causes of preterm mortality showed that little is known on the subject in low-resource settings. We searched PubMed, Excerpta Medica Database, ISI Web of Knowledge, and Cumulative Index to Nursing and Allied Health Literature for all studies on the causes of death in preterm infants, published from Jan 1, 2000, to June 30, 2016. We included studies of preterm and very low birthweight infants. We included the following search terms: “preterm birth” OR “prematurity” OR “very low birth weight” AND “illness” OR “morbidity” OR “complications” OR “death” OR “mortality”. To identify additional important articles, we used the related citations function in PubMed and hand-searched bibliographies of all articles that met our inclusion criteria, as well as review articles. We searched for English and French full articles and English or French abstracts of other articles written in other languages.

We identified 90 articles. We excluded 12 articles because they were not related to preterm infants but to premature adult deaths or to neonatal deaths broadly. 56 articles were excluded based on their title, mostly dealing with topics such as treatment of specific conditions, intervention trials, risk factors, and aetiological agents. We excluded an additional 14 articles that included mixed populations with older children. We finally identified eight articles, of which six were from high-income countries. There were two articles we reviewed from Brazil and South Africa (both of them middle-income countries) but in both cases, the data were collected retrospectively and the number of preterm infants involved was small. Overall, there is little evidence to inform policy makers on the major causes of preterm mortality. Because of this lack of evidence, we decided that there is a need to undertake a large-scale prospective study to establish the main causes of preterm mortality in a low-resource setting such as Ethiopia.

**Added value of this study**

Our study was a large, prospective study, involving multiple centres in a low-resource country. The study has clearly identified the major causes of mortality in preterm infants in a low-resource setting. The findings show that there are a handful of conditions for which we have effective interventions, albeit expensive. We need to work to develop affordable interventions through further research.

**Implications of all the available evidence**

The implications of the findings of our study are for national policy makers, programme managers, and implementing non-governmental organisations to take into consideration the main causes of preterm mortality when developing a package of interventions to reduce mortality in children younger than 5 years, or neonatal mortality in low-resource settings. For example, there was little attention to funding the management of respiratory distress syndrome in many of these settings. But this study revealed respiratory distress syndrome is responsible for 45% of cases of preterm mortality. We also know that there are specific interventions such as treatment with surfactant or bubble continuous positive airway pressure that are specific to respiratory distress syndrome. These interventions might not be affordable to scale in such settings and further research is required to develop simplified interventions. In addition, international support is required to support the scale-up of these interventions.

Historically, prematurity-related complications, referred to as prematurity, have been described as one entity among causes of mortality in children younger than 5 years and neonates.^7^, ^8^, ^9^, ^10^ However, preterm neonatal deaths could be related to either preterm-related complications (specific to the fact that the neonate is born preterm) or the deaths could be due to conditions such as congenital anomalies, asphyxia, or sepsis, which could also cause death in term infants. Common preterm causes of death include respiratory distress syndrome, necrotising enterocolitis, and intraventricular haemorrhage.^11^ The WHO International Classification of Diseases-10 (ICD-10) requests that clinicians do not enter prematurity as the main disease or condition in the fetus or infant unless it was the only fetal or infant condition known. There is a tendency in many settings to assign prematurity as a cause of death when further evidence to suggest a more definitive cause of death has not been actively sought.^12^

Despite progress made globally in identifying interventions to improve preterm outcomes,^13^ to our knowledge, few prospective studies have documented the distribution of the major causes of death among preterm births, particularly in countries such as Ethiopia. Capturing the events that led to death from both the maternal and fetal side informs the designs and development of interventions to reduce the burden of mortality,^12^ especially in resource-poor settings. In such settings, cause of death is often not recorded, and when recorded, estimates are often based on verbal autopsy data. Accurate data for the cause of death could facilitate the development of interventions and the prioritisation of scarce resources to implement the potential interventions at scale. To address the gaps in data for causes of death among preterm births, we aimed to establish the most common causes of death in preterm infants admitted to selected hospitals in Ethiopia on the basis of a standardised diagnostic protocol.

## Methods

### Study design and participants

The Study of Illness in Preterms (SIP) was a prospective, multicentre, cross-sectional, observational clinical study done in five hospitals in Ethiopia.

The study participants were preterm infants born at or referred within 7 days to the following five hospitals: in northwestern Ethiopia (Gondar University Hospital), in southwestern Ethiopia (Jimma University Hospital), and three hospitals in Addis Ababa (Ghandi Memorial Hospital, St Paul's Hospital Millennium Medical College, and Tikur Anbessa Hospital). A map of Ethiopia showing the study regions is included in the appendix.

All preterm, liveborn infants who were admitted to one of the study hospitals with an estimated gestational age of younger than 37 weeks were screened. The study hospital staff recruited preterm infants who were alive without any lower gestational age limit. Inclusion criteria were as follows: infant born at or transferred to one of the participating study hospitals; gestational age of younger than 37 weeks according to the algorithm using the three methods: ultrasound before 28 weeks of gestation, mother's report of last menstrual period, and the New Ballard score completed before 7 days of postnatal age; liveborn defined as infant with crying, breathing, or movement after delivery or Apgar of 1 or greater; infant age younger than 7 days at the time of screening; and parental consent given for study participation. Participants were excluded if delivery was a result of an induced abortion or gestational age could not be reliably established using the study criteria.

All eligible, liveborn babies were enrolled into the study. Infants who met the criteria were admitted regardless of whether the baby died before admission to the neonatal intensive care unit (NICU) or was discharged home without admission.

The study was approved by the institutional review board of each hospital and at the College of Health Sciences of the Addis Ababa University. Written consent for participation in the study was obtained from the parent or legal guardian, with a separate written consent for complete diagnostic autopsy and minimally invasive tissue sampling (MITS), in cases of death. Consent was obtained in English, Amharic, or Oromiffa languages, as appropriate. Confidentiality of the information was maintained.

All clinical procedures were done per hospital protocol. An informed consent form was developed for caretakers to read (or be read to them) so that the participants understood the implications of the research. For preterm infants who died, parents or caretakers were asked to complete a separate consent for post-mortem examinations of the whole body or samples of relevant tissue or fluids. The post-mortem examination could be complete diagnostic autopsy or MITS needle biopsy (or both) depending on the consent of parents or caretakers.

Details of the protocol for the SIP study have been published elsewhere.^14^

### Procedures and outcomes

Data were collected on maternal socioeconomic status, maternal and obstetric history, infant clinical conditions, imaging assessment, and microbiology of blood and cerebrospinal fluid specimens using predefined case report forms. Post-mortem examinations (both complete diagnostic autopsy and MITS) were done with additional consent from the parent or legal guardian. Following additional training, the MITS process was introduced in the second half of the study period. The clinical management of recruited patients followed the national guidelines developed by the Federal Ministry of Health of Ethiopia.^15^

To establish cause of death for each case, a panel consisting of international and national experts in neonatology, obstetrics, and pathology (including RP, SD, RLG, and HB) met face to face every 6 months and reviewed the deaths. For each death, the available clinical, laboratory, MITS, and autopsy data, including the sequence of clinical events during the infant's hospital stay, were available to panellists to establish the primary and contributory fetal or neonatal and maternal causes of mortality using a protocol.^14^

The panel was divided into pairs comprising an Ethiopian and international clinician; each pair established cause of death for a set of cases. Criteria were prospectively defined for the primary cause of death and prioritisation when more than one possible cause was present.^16^ The panel assigned the primary cause of death using ICD-10.^12^ The primary or underlying cause of death is the “disease or injury that initiated the train of events leading directly to death, or circumstances of accident or violence which produced the fatal injury”, unlike the immediate cause of death, which was defined as “the disease or complication which directly preceded or directly led to death”. If the two panel members were unable to reach consensus, the case was presented to the full expert panel for adjudication. If insufficient or conflicting information was present, the panel could decide that the cause could not be established with the available data. In addition to the primary fetal or neonatal cause of death, the panel also identified one or more neonatal and maternal conditions that contributed to each death.

Gestational age was established using a hierarchy of three methods: ultrasound before 28 weeks of gestation when available, mother's report of last menstrual period when judged reliable, and the New Ballard Score completed at younger than 7 days of age.^13^ The so-called best gestational age, which was used for the study purposes to document whether the infant met the criteria of preterm (<37 weeks gestation at delivery) was calculated using the hierarchy: (1) ultrasound at younger than 28 weeks of gestation; (2) ultrasound at more than 28 weeks of gestation with agreement of a reliable last menstrual period or New Ballard Score; (3) reliable last menstrual period and New Ballard Score; (4) if discrepancy between last menstrual period and New Ballard Score was greater than 2 weeks, the last menstrual period date was used; and (5) if no ultrasound and last menstrual period estimate was not reliable, New Ballard Score was used.^13^ If the infant was more than 37 weeks or if the gestational age could not be established with these methods, the infant was excluded from the study.

For follow-up of recruited infants, all hospitalised infants were assessed daily using a standardised tool and those at home were monitored once a week, either through a face-to-face meeting or by telephone. The research nurses and doctors underwent initial training on the case report forms, and their skills were reinforced every 3 months and with on-the-spot assessments. Each hospital team held once a week meetings to discuss and resolve challenges.

In the study hospital NICU, infants were evaluated once to twice per day by a research nurse who documented the findings on case report forms. A supervisor monitored the collection of data and biospecimen for laboratory evaluation. Relevant laboratory, radiology, and pathology personnel were informed about the samples sent. Visits to each hospital were done regularly by the investigators to check data quality and provide support as necessary.

### Statistical analysis

At each hospital, data were entered twice by different data clerks into study computers using the data management system developed for this study to control for errors. Data were transferred on a regular basis from each hospital's data management computer to the data centre at Addis Ababa University (Addis Ababa, Ethiopia), creating a complete data repository. Data were merged into one master dataset for analysis. Frequency of the findings from complete diagnostic autopsy and MITS was computed with 95% CIs using beta distribution to account for the finite population size.^17^

The sample size was calculated on the basis of the sample size for comparing proportions of causes of death. Assuming 20% of preterm deaths are due to one of the likely causes of preterm mortality, with a 4% precision and 95% level of confidence, 384 preterm deaths with confirmed cause of mortality were needed. Assuming attrition of 10–20%, and that 50% of participants would refuse to consent to post-mortem examinations, we estimated that between 770 and 960 preterm deaths (mean of 865) were required. With a case fatality rate of preterm admissions of 20%, we estimated that 4325 preterm admissions were required during the study period. Descriptive statistics were done using Stata version 14.2 (2017 package).

### Role of the funding source

The funder had no role in study design, data collection, data analysis, data interpretation, or writing of the report. The corresponding author had full access to all the data in the study and had final responsibility for the decision to submit for publication.

## Results

4919 preterm infants, of whom 3852 were admitted to the NICU, were recruited to the study over a period of nearly 2 years (from July 1, 2016, to May 31, 2018) in four of the hospitals (Gondar University Hospital, Jimma University Hospital, St Paul's Hospital Millennium Medical College, and Tikur Anbessa Hospital), and over a period of 7 months (from July 1, 2016, to Jan 31, 2017) in Ghandi Memorial Hospital.

**Figure 1:**
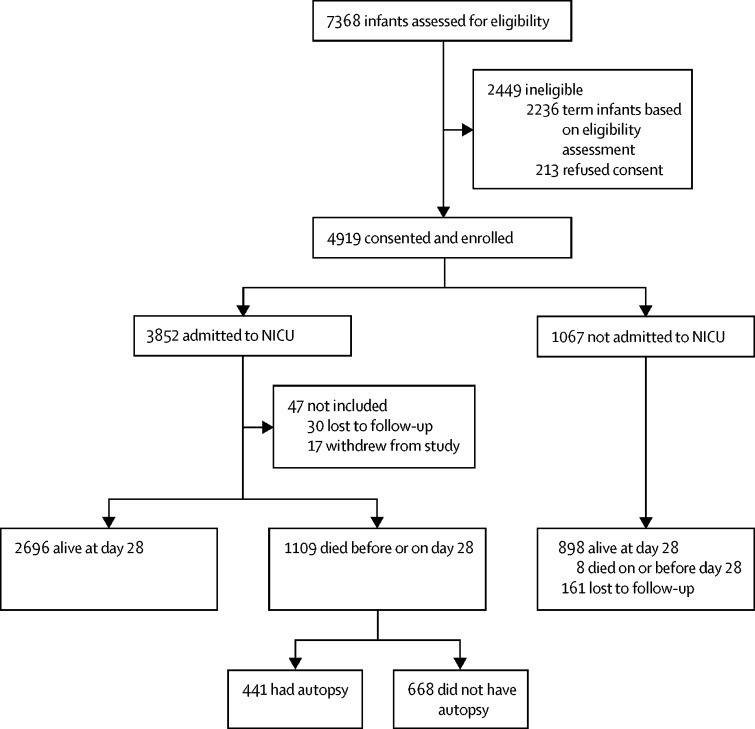
Study flow diagram NICU=neonatal intensive care unit.

Of the 4919 preterm births, 1117 (22·7%) died. Of the 3852 infants admitted to the NICU, 1109 (28·8%) died, 30 (0·8%) were lost to follow-up, and 17 (0·5%) withdrew from the study. Of the 1067 infants not admitted to the NICU, eight (0·7%) were reported dead at 28 days, 161 (15·1%) were lost to follow-up, and 898 were confirmed alive at day 28 (figure 1). The infants who withdrew from the study were discharged from the NICU against medical advice. The infants who were lost to follow-up were infants whose parents could not be accessed via the contact address or telephone number they provided at admission.

Baseline characteristics are shown in table 1. There were slightly more boys than girls among the enrolled infants. Preterm infants younger than 28 weeks accounted for about 104 (2·1%) of those enrolled. Of the NICU admissions, 1636 (42·5%) were 32–34 weeks of gestation and of those not admitted, 707 (66·2%) were 35–36 weeks of gestation. 3459 (70·1%) of all enrolled infants were born in the study hospitals whereas 1191 (24·2%) were transferred from another health facility and 278 (5·7%) were home deliveries. 1293 (36·2%) of NICU admissions were multiple births (table 1).

**Table 1:** Characteristics of enrolled infants by status at admission and outcome

	**Total enrolled (N=4919)**	**Admitted toNICU (n=3852)**	**Not admitted to NICU (n=1067)**	**Died (n=1109)**
**Sex**				
Male	2543 (51·7%)	2032 (52·7%)	511 (48·1%)	598 (53·9%)
Female	2315 (47·1%)	1767 (45·9%)	548 (51·4%)	495 (44·6%)
Missing data	61 (1·2%)	53 (1·4%)	8 (7·5%)	16 (1·4%)
**Gestational age (weeks)**				
<28	104 (2·1%)	104 (2·7%)	0	89 (8·0%)
28 to 31	952 (19·4%)	931 (24·2%)	21 (2·0%)	512 (46·2%)
32 to 34	1975 (40·2%)	1636 (42·5%)	339 (31·8%)	349 (31·5%)
35 to <37	1888 (38·3%)	1181 (30·6%)	707 (66·2%)	159 (14·3%)
**Birthweight (g)**				
<1000	165 (3·4%)	165 (4·3%)	0	137 (12·3%)
1000 to <1500	1013 (20·6%)	982 (25·5%)	31 (2·9%)	482 (43·4%)
1500 to <2000	1896 (38·5%)	1478 (38·4%)	418 (39·2%)	314 (28·3%)
≥2000	1751 (35·6%)	1147 (29·8%)	604 (56·6%)	155 (14·0%)
Missing	94 (1·9%)	80 (2·1%)	14 (1·3%)	22 (2·0%)
**Study hospital**				
Ghandi Memorial Hospital	424 (8·6%)	382 (9·9%)	42 (3·9%)	92 (8·3%)
Gondar University Hospital	1019 (20·7%)	891 (23·1%)	128 (12·0%)	257 (23·2%)
Jimma University Hospital	692 (14·1%)	526 (13·7%)	166 (15·6%)	188 (17·0%)
St Paul's Hospital	1629 (33·1%)	1041 (27·0%)	588 (55·1%)	298 (26·9%)
Tikur Anbessa Hospital	1155 (23·5%)	1012 (26·3%)	143 (13·4%)	274 (24·7%)
**Place of birth**				
Study hospital	3450 (70·1%)	2686 (69·7%)	764 (71·6%)	886 (79·9%)
Non-study hospital	1191 (24·2%)	982 (25·5%)	209 (19·6%)	157 (14·2%)
Home	278 (5·7%)	184 (4·8%)	94 (8·8%)	66 (5·9%)
**Singleton or multiple births**				
Single	3140 (63·8%)	2559 (66·4%)	581 (54·5%)	784 (70·7%)
Twins	1656 (33·7%)	1187 (30·9%)	469 (44·0%)	290 (26·1%)
Triplets	115 (2·3%)	98 (2·5%)	17 (1·5%)	32 (2·9%)
Quadruplets or more	8 (0·2%)	8 (0·2%)	0	3 (0·3%)

Data are n (%). NICU=neonatal intensive care unit.

As shown in table 1, among the 1109 deaths of infants who were in the NICU, those born at younger than 28 weeks of gestation contributed to only 8·0% of these deaths. However, among the same gestational age categories, mortality was highest among those born at younger than 28 weeks of gestational age. Of the infants enrolled in the study, mortality was 85·6% (89 of 104) for infants younger than 28 weeks of gestation, 53·7% (512 of 952) for infants of 28 to less than 32 weeks of gestation, 17·7% (349 of 1975) for infants of 32 to less than 35 weeks gestation, and 8·4% (159 of 1888) for infants of 35 to less than 37 weeks of gestation. Similarly, mortality was inversely associated with birthweight with the death of 137 (83·0%) of 165 preterm infants who weighed less than 1000 g, 482 (47·6%) of 1013 infants who weighed between 1000 g to less than 1500 g, 314 (16·6%) of 1896 infants who weighed 1500 g to less than 2000 g, and 155 (8·8%) of 1751 infants who weighed 2000 g or more.

Table 2 summarises the main investigations done for common admission diagnoses. The low proportion of investigations done for these diagnoses shows that clinical investigations were frequently not done.

**Table 2:** Investigations at admission to support diagnosis by the expert panel

		**Infants (%)**
Respiratory distress syndrome		1721/3852 (45%)
	Chest x-ray	274/1721 (16%)
Sepsis		1422/3852 (37%)
	White blood count	1243/1422 (87%)
	C-reactive protein	670/1422 (47%)
	Blood culture	744/1422 (52%)
Pneumonia		274/3852 (7%)
	Chest x-ray	79/274 (29%)
Intraventricular haemorrhage		57/3852 (1%)
	Cranial ultrasound	38/57 (67%)
Necrotising enterocolitis		155/3852 (4%)
	Abdominal x-ray	24/155 (16%)

Data are n (%). Multiple investigations possible for each individual.

Table 3 summarises the frequency of complete diagnostic autopsy and MITS done for the deaths. Among the 1109 deaths, complete diagnostic autopsy was done in 441 (39·8%) over the whole period of the study, whereas MITS was done in 126 (11·4%) of the deaths in the last half of the study period. Among the 441 deaths in which the infant had a complete diagnostic autopsy, 241 (54·6%) were boys, 7·9% were younger than 28 weeks of gestation, 213 (48·3%) were between 28 to 32 weeks of gestation, and 134 (30·4%) were between 32 to 34 weeks of gestation. This result is similar to the distribution of the deaths shown in table 1. On the basis of complete diagnostic autopsy, respiratory distress syndrome contributed to 50·8% (95% CI 47·1–54·5); sepsis to 20·4% (17·6–23·6), and asphyxia to 15·0% (12·5–17·8) of the deaths. On the basis of MITS, respiratory distress syndrome contributed to 48·4% (95% CI 39·9–56·9), sepsis to 17·5%, (11·6–24·8), and asphyxia to 15·9% (10·3–23·0).

**Table 3:** Post-mortem diagnosis

	**Complete diagnostic autopsy**	**Minimally invasive tissue sampling**
All deaths (N=1109)	441 (39·8%)	126 (11·4%)
Respiratory distress syndrome (n=502, 45·3%)	224 (50·8%, 47·09–54·49)	61 (48·4%, 39·94–56·95)
Sepsis (n=289, 26·1%)	90 (20·4%, 17·56–23·57)	22 (17·5%, 11·64–24·78)
Pneumonia (n=33, 3·0%)	19 (4·3%, 2·99–6·12)	4 (3·2%, 1·01–7·65)
Intraventricular haemorrhage (n=12, 1·1%)	4 (0·9%, 0·40–1·99)	3 (2·4%, 0·60–6·54)
Necrotising enterocolitis (n=9, 0·8%)	0	0
Asphyxia (n=151, 13·6%)	66 (15·0%, 12·48–17·82)	20 (15·9%, 10·32–23·00)
Others (n=113, 10·2%)	38 (8·6%, 6·72–10·96)	16 (12·7%, 7·74–19·39)

Data are n (%, 95% CI). Complete diagnostic autopsy or minimally invasive tissue sampling done among neonatal intensive care unit deaths to support cause of death determination by the expert panel.

The distribution of the primary causes of death is shown in table 4. The panel of experts reviewed 1109 cases. Of these, cause of death was established for 1104 individuals, but five individuals did not have a cause established other than prematurity. Of the 20 potential causes of death that were coded in the case report forms, respiratory distress syndrome, sepsis, pneumonia, asphyxia, intraventricular haemorrhage, apnoea, meningitis, and congenital anomalies contributed to 1022 (92%) of all deaths.

**Table 4:** Distribution of primary cause of death of preterm infants by gestational age

	All infants*	**<28 weeks**†	**28–31 weeks**†	**32–34 weeks**†	**35 to <36 weeks**†
Respiratory distress syndrome	502 (45·3%)	53 (10·6%)	264 (52·6%)	150 (29·9%)	35 (7·0%)
Sepsis	289 (26·1%)	15 (5·2%)	121 (41·9%)	99 (34·3%)	54 (18·7%)
Pneumonia	33 (3·0%)	3 (9·1%)	14 (42·4%)	11 (33·3%)	5 (15·2%)
Meningitis	9 (0·8%)	0	1 (11·1%)	2 (22·2%)	6 (66·7%)
Congenital anomalies	38 (3·4%)	0	9 (23·7%)	13 (34·2%)	16 (42·1%)
Asphyxia	151 (13·6%)	14 (9·3%)	66 (43·7%)	43 (28·5%)	28 (18·5%)
Intraventricular haemorrhage	12 (1·1%)	0	5 (41·7%)	6 (50·0%)	1 (8·3%)
Apnoea	16 (1·4%)	4 (25·0%)	7 (43·8%)	4 (25·0%)	1 (6·3%)
Necrotising enterocolitis	9 (0·8%)	0	2 (22·2%)	5 (55·6%)	2 (22·2%)
Others	50 (4·5%)	0	23 (46·0%)	15 (30·0%)	12 (2·0%)

Data are n (%). N=1109. One primary cause of death was allowed per individual.

* Denominator is total number of infants who died (N=1109).

† Denominator is total number of infants who died of that particular cause.

**Figure 2:**
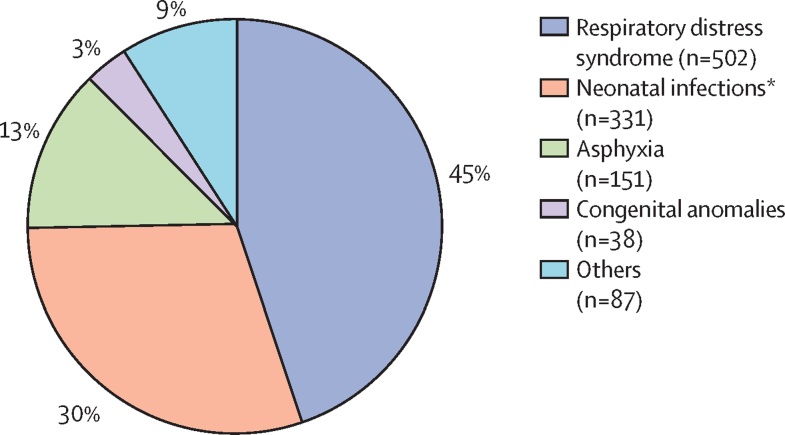
Distribution of primary cause of death of preterm infants N=1109. The four most common causes of death are shown and the less common causes are grouped as others. *Sepsis, pneumonia, and meningitis.

As shown in table 4, the main primary causes of death were respiratory distress syndrome, sepsis, pneumonia, and meningitis (referred here in combination as neonatal infections, which accounted for 331 [29·8%] of 1109 deaths) and asphyxia. To enable comparison with global estimates, for example, by the UN Inter-agency Group for Child Mortality Estimation,^4^ we used respiratory distress syndrome, neonatal infections, asphyxia, and congenital anomalies and collapsed the others under other causes (figure 2). Respiratory distress syndrome, neonatal infections, and asphyxia together were responsible for 984 (88·7%) of all deaths. Congenital anomalies were responsible for 38 (3·4%) of the deaths. Table 4 shows the distribution of primary causes of death by gestational age categories; the distribution of primary causes of death by hospital, by birthweight category, and causes of death when primary and contributory causes are combined are shown in the appendix.

There were multiple neonatal and maternal contributory causes of death (Table 5, Table 6). Hypothermia was the most common neonatal contributory cause; present in 770 (69·4%) of all deaths. Other neonatal contributory causes included respiratory distress syndrome, apnoea, sepsis, anaemia, and hyperbilirubinaemia. Pre-eclampsia and eclampsia (229 [20·6%] 1109) were the most common maternal risk factors that might have contributed to preterm death. Other maternal contributory factors included antenatal haemorrhage, chorioamnionitis, anaemia, premature rupture of membranes, breech presentation, prolonged labour, diabetes, heart disease, HIV, hepatitis, and malaria. The contributory causes of death by hospital and birthweight category are shown in the appendix.

**Table 5:** Neonatal contributory causes of death of preterm infants by gestational age

	**<28 weeks**	**28 to 31 weeks**	**32 to 34 weeks**	**35 to <36 weeks**	**All (N)**
Hypothermia	71 (9·2%)	369 (47·9%)	237 (30·8%)	93 (12·1%)	770
Apnoea	38 (10·6%)	185 (51·4%)	109 (30·3%)	28 (7·8%)	360
Respiratory distress syndrome	29 (8·4%)	166 (48·3%)	104 (30·2%)	45 (13·1%)	344
Sepsis	14 (8·5%)	73 (44·5%)	52 (31·7%)	25 (15·2%)	164
Hyperbilirubinaemia (jaundice)	10 (8·2%)	43 (35·2%)	44 (36·1%)	25 (20·5%)	122
Anaemia	10 (9·8%)	45 (44·1%)	32 (31·4%)	15 (14·7%)	102
Asphyxia	7 (7·1%)	47 (47·5%)	34 (34·3%)	11 (11·1%)	99
Hypoglycaemia	4 (5·4%)	30 (40·5%)	26 (35·1%)	14 (18·9%)	74
Intraventricular haemorrhage	6 (8·8%)	37 (54·4%)	21 (30·9%)	4 (5·9%)	68
Pneumonia	5 (8·9%)	19 (33·9%)	19 (33·9%)	13 (23·2%)	56

Data are n (%). N=1109. More than one contributory condition is possible so percentages are calculated across gestational age categories. In some cases, conditions that were often a primary cause of death such as respiratory distress syndrome were present but deemed by the panel to be a contributory cause.

**Table 6:** Maternal contributory causes of death of preterm infants by gestational age

	**<28 weeks**	**28 to 31 weeks**	**32 to 34 weeks**	**35 to <36 weeks**	**All (N)**
Pre-eclampsia and eclampsia	11 (4·8%)	117 (51·1%)	90 (39·3%)	11 (4·8%)	229
Antenatal hemorrhage	10 (11·8%)	46 (54·1%)	20 (23·5%)	9 (10·6%)	85
Maternal fever	3 (7·7%)	15 (38·5%)	18 (46·2%)	3 (7·7%)	39
Chorioamnionitis	7 (13·5%)	26 (50·0%)	15 (28·9%)	4 (7·7%)	52
Cord prolapsed	0	4 (36·0%)	3 (27·3%)	4 (36·4%)	11
Signs of fetal distress	0	5 (55·6%)	2 (22·2%)	2 (22·2%)	9
Obstructed labour	0	2 (50·0%)	0	2 (50%)	4
Other	21 (7·5%)	126 (44·8%)	95 (33·8%)	39 (13·9%)	281

Data are n (%). N=1109. More than one contributory condition is possible so percentages are calculated across gestational age categories.

## Discussion

Our study found that only a few conditions contributed to the majority of preterm deaths. Respiratory distress syndrome alone contributed to 45% of the primary causes of preterm death. Sepsis, meningitis, and pneumonia combined contributed to nearly 30% of the primary causes of preterm death followed by asphyxia, which contributed to about 14% of deaths. There was generally more than one contributory cause identified by the panel. Hypothermia was the most common contributory cause of mortality; 69% of all deaths presented with hypothermia.

Mortality was highest among preterm infants younger than 28 weeks of gestation (8%) or with a birthweight of less than 1000 g (12%), even though the absolute number of deaths contributed a low proportion of the overall mortality. An Iranian study^18^ showed that mortality was highest among infants less than 28 weeks of gestation with the most common cause of neonatal death being respiratory distress syndrome (73·8%). A retrospective study^19^ of 129 preterm infants from an NICU in Trinidad and Tobago showed that extremely low birthweight (ie, less than 1000 g) accounted for 75% of the deaths and 31% had sepsis or infections and pneumonia as a cause of death on their death certificate. In a retrospective study among late preterm infants (34–37 weeks) in Turkey,^20^ the main causes of death were respiratory distress (62%), congenital anomalies (61%), sepsis (49%), and asphyxia (25%). Our study showed that congenital anomalies contributed to only 3·4% of the overall mortality. Among 284 preterm infants admitted to the NICU of a university hospital in Londrina, Brazil, with birthweight less than 1500 g or gestational age of younger than 31 weeks (or both), the neonatal mortality was 23·2%, varying according to birthweight.^21^

In contrast to the 55% mortality of preterm infants born before 32 weeks gestation in our study, in developed countries such as in Australia, among infants younger than 32 weeks gestation admitted to ten NICUs, mortality was 7·7%.^22^ In Switzerland, using prospectively collected data from a 10-year cohort of nearly 7000 very-low gestational age infants born at younger than 32 weeks of gestation, mortality was 15%.^23^

Our study had standardised operating procedures and standardised case report forms that were completed by research nurses and doctors who had undergone initial training on the case report forms and on-the spot assessments. Monitoring visits were done to sites by external and internal senior researchers to ensure good quality data. The decision on causes of death was made based on data collected on maternal and obstetric risk factors, clinical observations made at admission, and the documented subsequent clinical sequence of events. When available, investigations including haematology, microbiology, imaging, and a complete diagnostic autopsy or MITS were used. Preterm infants can have multiple morbidities.^24^ However, differentiating clinical diagnosis of the common conditions such as sepsis, respiratory distress syndrome, pneumonia, or even intraventricular haemorrhage has been difficult. Establishing a single cause of death when multiple causes play a role was challenging and subjective. Complete diagnostic autopsy was available in only 40% of the deaths potentially introducing bias towards a selected population. The other limitation of our study was that imaging, and in particular chest x-rays, were done infrequently, again potentially introducing bias. Despite these limitations, all attempts were made to reduce bias using standardised definitions and protocols based on a multilayered approach to classification of ICD-10, Perinatal Period.^12^

Interventions to improve preterm birth outcomes such as antenatal corticosteroids to improve neonatal lung maturation, thermal care for preterm infants, essential newborn care including resuscitation capacity, and continuous positive airway pressure for respiratory distress syndrome are recommended globally.^12^ However, the coverage of these interventions has often been low and of poor quality.^25^, ^26^ In this study, corticosteroids were used for only 700 (31·2%) mothers of 2243 preterm babies whose gestational age was between 24 to 34 weeks. In Ghana, the overall prevalence of adequate newborn care comprising good cord care, optimal thermal care, and good neonatal feeding practices was only 15·8%.^27^ In a survey of 98 health facilities in Ethiopia, only 27% had a heat source in their delivery room, about 12% did not assess the babies breathing at birth, and 66% had the basic equipment for neonatal resuscitation.^28^ The coverage of such interventions, including antibiotics for sepsis and resuscitation at birth, need to be scaled up along with improvement in supportive infrastructure and general newborn care including capacity building of nursing staff and clinicians, as well as optimal breastfeeding and parenteral nutrition.^29^ Given that respiratory distress syndrome was responsible for 45% of preterm mortality, interventions specific to respiratory distress syndrome such as continuous positive airway pressure, and blended oxygen need to be available to have an impact on the high neonatal mortality observed in low-income and middle-income countries.

In conclusion, our findings suggest respiratory distress syndrome, sepsis, and asphyxia are the main causes of preterm mortality. Our study has highlighted the challenges that exist in the classification of cause of death of preterm infants and the need to strengthen capacity of health professionals on the classification of cause of death previously lumped as prematurity. Our study points to the need for policy change to invest in interventions targeting the major causes of preterm mortality to meet the SDG target of reducing neonatal mortality to less than 12 per 1000.^30^ Further research is called for to confirm the findings of our study. We also recommend research to develop effective and affordable interventions to tackle the major causes of preterm death.
