# Supplementary material for: Major causes of death in preterm infants in selected hospitals in Ethiopia (SIP): a prospective, cross-sectional, observational study
Source: Lancet Glob Health. 2019 Jul 11;7(8):e1130–8. doi: 10.1016/S2214-109X(19)30220-7 (PMC6639243; doi:10.1016/S2214-109X(19)30220-7)

THE LANCET
Global Health

## Supplementary appendix

This appendix formed part of the original submission and has been peer reviewed. We post it as supplied by the authors.

Supplement to: Muhe LM, McClure EM, Nigussie AK, et al. Major causes of death in preterm infants in selected hospitals in Ethiopia (SIP): a prospective, cross-sectional, observational study. *Lancet Glob Health* 2019; **7:** e1130–38.

**Appendix 1: Extra slides showing distribution of causes of death (primary, contributory or combined) by study hospitals, and by birth weight**

Table 7: Distribution of primary causes of death by study hospitals

| Primary COD (n%) | GMH | GUH | JUH | SPH | TAH | All |
|---|---|---|---|---|---|---|
| RDS | 54(10.7) | 115(22.9) | 76(15.1) | 141(28.1) | 116(23.1) | 502 |
| Sepsis | 16(5.5) | 70(24.2) | 63(21.8) | 90(31.1) | 50(17.3) | 289 |
| Pneumonia | 1(3.0) | 4(12.1) | 5(15.2) | 10(30.3) | 13(39.4) | 33 |
| Meningitis | 0 | 1(11.1) | 3(33.3) | 3(33.3) | 2(22.2) | 9 |
| Congenital anomalies | 0 | 4(10.5) | 4(10.5) | 3(7.9) | 27(71.1) | 38 |
| Asphyxia | 15(9.9) | 39(25.8) | 20(13.2) | 34(22.5) | 43(28.5) | 151 |
| IVH | 0 | 1(8.3) | 2(16.7) | 5(41.7) | 4(33.3) | 12 |
| Apnoea | 1(6.3) | 6(37.5) | 5(31.3) | 3(18.8) | 1(6.3) | 16 |
| NEC | 0 | 6(66.8) | 1(11.1) | 0 | 2(22.2) | 9 |
| Others | 5 (10) | 11 (22) | 9 (18) | 9 (18) | 16 (32 | 50 |

Table 8: Distribution of contributory causes of death by study hospitals

| Neonatal contributory causes (n %) | GMH | GUH | JUH | SPH | TAH | All |
|---|---|---|---|---|---|---|
| Hypothermia* | 77(10.0) | 164(21.4) | 117(15.2) | 216(28.1) | 194(25.5) | 768 |
| Apnoea | 43(11.9) | 81(22.5) | 23(6.4) | 142(39.4) | 71(19.7) | 360 |
| Respiratory Distress Syndrome | 30(8.9) | 64(18.9) | 48(14.2) | 111(32.8) | 85(25.2) | 338 |
| Sepsis | 11(6.9) | 61(38.1) | 15(9.4) | 29(18.1) | 44(27.5) | 160 |
| Hyperbilirubinemia (Jaundice) | 3(2.5) | 44(36.1) | 22(18.0) | 33(27.1) | 20(16.4 | 122 |
| Anemia | 5(4.9) | 36(35.3) | 20(19.6) | 15(14.7) | 26(25.5) | 102 |
| Asphyxia | 7(7.1) | 23(23.2) | 13(13.1) | 27(27.3) | 27(27.3) | 99 |
| Hypoglycemia | 0 | 11(14.7) | 22(29.3) | 32(42.7) | 10(13.3) | 75 |
| Intraventricular Hemorrhage | 7(10.3) | 18(26.5) | 6(8.8) | 23(33.8) | 14(20.6) | 68 |
| Pneumonia | 2(3.6) | 7(12.5) | 4(7.1) | 27(48.2) | 16(28.6) | 56 |

*multiple conditions are possible

Table 9: Contribution of maternal conditions by study hospitals

| Maternal conditions n(%) | GMH | GUH | JUH | SPH | TAH | All |
|---|---|---|---|---|---|---|
| Pre-eclampsia* and eclampsia | 21(8.1) | 36(13.9) | 27(10.4) | 94(36.3) | 81(31.3) | 259 |
| Antenatal hemorrhage | 5(5.9) | 31(36.5) | 24(28.2) | 11(12.9) | 14(16.5) | 85 |
| Maternal fever | 4(10.3) | 25(64.1) | 4(10.3) | 5(12.8) | 1(2.6) | 39 |
| Chorioamnionitis | 2(3.9) | 11(21.2) | 14(26.9) | 18(34.6) | 7(13.5) | 52 |
| Cord prolapse | 2(18.2) | 0 | 2(18.2) | 4(36.4) | 3(27.2) | 11 |
| Signs of fetal distress | 1(11.1) | 2(22.2) | 2(22.2) | 2(22.2) | 2(22.2) | 9 |
| Obstructed labor | 0 | 1(25) | 0 | 2(50) | 1(25) | 4 |
| Others | 34(12.1) | 46(16.4) | 44(15.7) | 93(33.1) | 61(21.7) | 281 |

Table 10: Distribution of primary causes of death by birth weight categories

| Primary COD n(%) | >1000 | 1000-1500 | 1500-2000 | >=2000 | Missing | All (N%) |
|---|---|---|---|---|---|---|
| RDS | 84(16.7) | 236(47.0) | 136(27.1) | 37(7.4) | 9(1.8) | 502 |
| Sepsis | 18(6.2) | 124(42.9) | 94(32.5) | 18(6.2) | 7(2.4) | 289 |

| | | | | | | |
|---|---|---|---|---|---|---|
| Pneumonia | 0 | 17(51.5) | 5(15.2) | 10(30.3) | 1(3.0) | 33 |
| Meningitis | 0 | 2(22.2) | 3(33.3) | 4(44.4) | 0 | 9 |
| Mal/chromo | 3(7.9) | 11(29.0) | 11(29.0) | 12(31.6) | 1(2.6) | 38 |
| Asphyxia | 25(16.6) | 54(35.8) | 38(25.2) | 30(19.9) | 4(2.7) | 151 |
| IVH | 1(8.3) | 6(50) | 2(16.7) | 3(25) | 0 | 12 |
| Apnoea | 3(18.8) | 5(31.3) | 6(37.5) | 2(12.5) | 0 | 16 |
| NEC | 0 | 5(55.6) | 3(33.3) | 1(11.1) | 0 | 9 |
| Others | 3(6) | 22(44) | 16(32) | 9(18) | 0 | 50 |

Table 11: Distribution of contributory causes of death by birth weight categories

| Contributory COD n(%) | >1000 | 1000-1500 | 1500-2000 | >=2000 | Missing | All |
|---|---|---|---|---|---|---|
| Hypothermia* | 110(14.3) | 335(43.6) | 209(27.2) | 96(12.5) | 18(2.3) | 768 |
| Apnoea | 56(15.6) | 165(45.8) | 93(25.8) | 40(11.1) | 6(1.7) | 360 |
| Respiratory Distress Syndrome | 45(13.3) | 144(42.6) | 94(27.8) | 46(13.6) | 9(2.7) | 338 |
| Sepsis | 17(10.6.) | 77(48.1) | 43(26.9) | 16(10) | 7(4.4) | 160 |
| Hyperbilirubinemia(Jaundice) | 7(5.7) | 49(40.2) | 46(37.7) | 19(15.6) | 1(0.8) | 122 |
| Anemia | 14(13.7) | 41(40.2) | 31(30.4) | 16(15.9) | 0 | 102 |
| Asphyxia | 12(12.1) | 50(50.5) | 25(25.3) | 9(9.1) | 1(1.0) | 99 |
| Hypoglycemia | 7(9.3) | 29(38.7) | 26(34.7) | 12(16) | 1(1.3) | 75 |
| Intraventricular Hemorrhage | 9(13.3) | 35(51.5) | 18(26.5) | 5(7.4) | 1(1.5) | 68 |
| Pneumonia | 7(12.5) | 22(39.3) | 19(33.9) | 7(12.5) | 1(1.8) | 56 |

Table 12: Contribution of maternal conditions by birth weight categories

| Maternal conditions n(%) | >1000 | 1000-1500 | 1500-2000 | >=2000 | missing | All |
|---|---|---|---|---|---|---|
| Pre-eclampsia* and eclampsia | 41(15.8) | 111(42.9) | 66(25.5) | 39(15.1) | 3(1.2) | 259 |
| Antenatal | 14(16.2) | 39(45.9) | 21(24.7) | 10(11.8) | 1(1.2) | 85 |

| | | | | | | |
|---|---|---|---|---|---|---|
| hemorrhage | | | | | | |
| Maternal fever | 3(7.7) | 18(46.2) | 15(38.5) | 2(5.1) | 1(2.6) | 39 |
| Chorioamnionitis | 7(13.5) | 24(46.2) | 13(25) | 6(11.5) | 2(3.8) | 52 |
| Cord prolapsed | 2(18.2) | 4(36.4) | 3(27.3) | 2(18.2) | 0 | 11 |
| Signs of fetal distress | 0 | 5(55.6) | 1(11.1) | 3(33.3 | 0 | 9 |
| Obstructed labor | 0 | 3(75) | 1(25) | 0 | 0 | 4 |
| Others | 113(40.2) | 72(25.6) | 40(14.2) | 50(17.8) | 62.1 | 281 |

Table 13: Causes of death when primary and contributory causes are combined

*Note multiple causes are possible for contributory causes

| Common Causes of Death | Primary COD | Contributory COD* | Combined |
|---|---|---|---|
| RDS | 502 | 768 | 840 |
| Sepsis | 289 | 360 | 449 |
| Pneumonia | 33 | 338 | 89 |
| Meningitis | 9 | 160 | 20 |
| Cong anomalies | 38 | 122 | 71 |
| Asphyxia | 151 | 102 | 248 |
| IVH | 12 | 99 | 80 |
| Apnoea | 16 | 75 | 376 |
| NEC | 9 | 68 | 34 |
| Others | 50 | 56 | 1196 |

Figure 1: Map of Ethiopia showing where the study hospitals are located (one in North Gondar in Amhara regional state, one in Jimma in Oromia regional state and 3 in Addis Ababa)

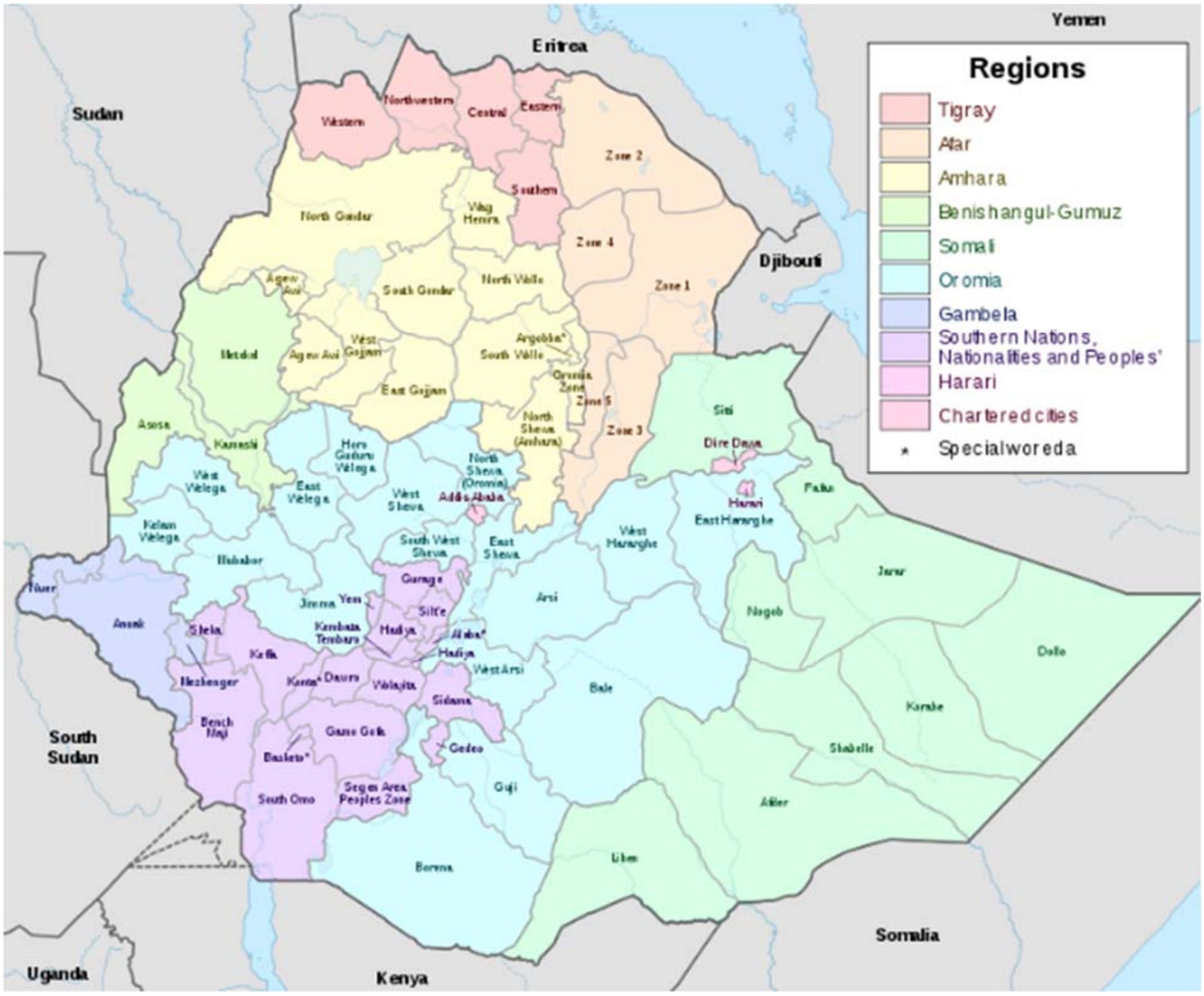

Supplement: Supplementary appendix [file mmc1.pdf]
